# A Deep Learning Ensemble Method to Assist Cytopathologists in Pap Test Image Classification

**DOI:** 10.3390/jimaging7070111

**Published:** 2021-07-09

**Authors:** Débora N. Diniz, Mariana T. Rezende, Andrea G. C. Bianchi, Claudia M. Carneiro, Eduardo J. S. Luz, Gladston J. P. Moreira, Daniela M. Ushizima, Fátima N. S. de Medeiros, Marcone J. F. Souza

**Affiliations:** 1 Departamento de Computação, Universidade Federal de Ouro Preto (UFOP), Ouro Preto 35400-000, Brazil; andrea@ufop.edu.br (A.G.C.B.); eduluz@ufop.edu.br (E.J.S.L.); gladston@ufop.edu.br (G.J.P.M.); marcone@ufop.edu.br (M.J.F.S.); 2 Departamento de Análises Clínicas, Universidade Federal de Ouro Preto (UFOP), Ouro Preto 35400-000, Brazil; mariana.trevisan@aluno.ufop.edu.br (M.T.R.); carneirocm@ufop.edu.br (C.M.C.); 3 Computational Research Division, Lawrence Berkeley National Laboratory, Berkeley, CA 94720, USA; dushizima@lbl.gov; 4 Berkeley Institute for Data Science, University of California, Berkeley, CA 94720, USA; 5 Bakar Computational Health Sciences Institute, University of California, San Francisco, CA 94143, USA; 6 Departamento de Engenharia de Teleinformática, Universidade Federal do Ceará (UFC), Fortaleza 60455-970, Brazil; fsombra@ufc.br

**Keywords:** deep learning, ensemble of classifiers, cervical cancer, Pap smear, images classification

## Abstract

In recent years, deep learning methods have outperformed previous state-of-the-art machine learning techniques for several problems, including image classification. Classifying cells in Pap smear images is very challenging, and it is still of paramount importance for cytopathologists. The Pap test is a cervical cancer prevention test that tracks preneoplastic changes in cervical epithelial cells. Carrying out this exam is important in that early detection. It is directly related to a greater chance of curing or reducing the number of deaths caused by the disease. The analysis of Pap smears is exhaustive and repetitive, as it is performed manually by cytopathologists. Therefore, a tool that assists cytopathologists is needed. This work considers 10 deep convolutional neural networks and proposes an ensemble of the three best architectures to classify cervical cancer upon cell nuclei and reduce the professionals’ workload. The dataset used in the experiments is available in the Center for Recognition and Inspection of Cells (CRIC) Searchable Image Database. Considering the metrics of precision, recall, F1-score, accuracy, and sensitivity, the proposed ensemble improves previous methods shown in the literature for two- and three-class classification. We also introduce the six-class classification outcome.

## 1. Introduction

Pap testing can detect cervical cancer upon tracks pre-neoplastic changes in cervical epithelial cells. The traditional method of Pap test is the conventional cytology. In the process of Pap smear collection, the professional responsible for the collection exposes the cervix with the introduction of a speculum. Then, using a spatula, the professional performs the collection of the cervical cells. The cells are put on a glass slide—called a smear—and are sent for analysis in laboratories that specialize in cytopathology.

There are approximately 15,000 fields per image (40× objective) on one slide with a collection of cellular samples from a conventional examination that must be manually analyzed under an optical microscope by a qualified cytopathologist. In certain countries, the workload can reach 100 smears per day. Furthermore, there is another recommendation that at least two professionals analyze the same smear to avoid false negatives. The large volume of smears analyzed on the same day often causes errors in scrutiny and diagnostic interpretation due to the professionals’ physical and mental fatigue. The procedure also requires much technical knowledge on the specialist’s part, which reduces the number of people who can perform it and increases the examination cost due to the necessary specialized labor costs.

Despite the use of the Pap smear test since the 1940s, the method has inherent limitations. Errors that lead to false positives (cases in which a lesion is mistakenly found) and false negatives (cases in which an existing lesion is not detected) can occur due to problems in all examination stages. These stages range from cytological material collection to lesion interpretation. Errors in the manual process of microscopic analysis of smears can reach 62% [1,2,3,4]. These errors directly impact the clinical patient’s conduct. In the first case, the patient is left without assistance and can silently develop cervical cancer, as the professional did not identify the alterations during the exam. In the second case, the patient develops feelings of anguish and is submitted to unnecessary procedures, as the professional misclassified that lesion, resulting in erroneous clinical behavior.

With the evolution of technologies, several systems that use computational algorithms to automatically analyze cell images have been developed in order to improve screening efficiency and accuracy. Some authors, such as Silva et al. [5] and Isidoro et al. [6], used traditional machine learning techniques (a support vector machine and handcrafted features) to perform the cell classification, while others employed convolutional neural networks to perform the classification [7,8,9].

Despite the number of works concerned with this problem, William et al. [10] demonstrated weaknesses in the algorithms available for the automatic detection of preneoplastic lesions in Papanicolaou images, which resulted in low classification accuracy in the different preneoplastic classes. In addition, they showed that the literature works performed the validation of most of the algorithms on small datasets with synthetic images, which were derived from cytology smears in liquid-based cytology; that is, the results are not reproducible in a real environment using conventional cytology [10,11].

Thus, this work aims to compare state-of-the-art convolutional neural network architectures to analyze the classification of real images of cervical cells obtained from conventional Pap smears to improve the reliability of the test results with the goal of reproducibility in a real environment. We can summarize the contributions of this work as follows:

- Proposal of a simple yet efficient ensemble method for improving the classification task;
- A data augmentation methodology to compensate for dataset imbalance;
- Classification analyses of different numbers of classes (two, three, and six) and their benefits;
- Investigation of the EfficientNets models, which are currently state of the art for the ImageNet dataset and have not yet been investigated for the cervical cell classification problem;
- Introduction of the results for six-class classification;
- State-of-the-art results for the cervical cell collection of the Center for Recognition and Inspection of Cells (CRIC), CRIC Cervix. Searchable Image Database [12].

As quality protocols are used in smear reviews to avoid errors, one of our methodology’s potential applications is to review smears that have already undergone a specialist’s first reading. Another application is to use the proposed method in the first reading of the smear. In this way, their use would reduce the analysis time.

Around 90 to 97% of Pap smears manually analyzed by cytopathologists are normal (without injury). Therefore, as proposed in this work, a cell screening methodology would have a wide application in professionals’ routines. The goal is to exclude normal cells and present only those with cytopathological changes to professionals, who will then diagnose. A cytopathologist can analyze up to 100 smears daily, which further justifies applying a screening methodology to support, facilitate, and improve their decisions.

## 2. Related Works

The automatic classification of cervical lesions is a challenging task in machine learning. Many studies apply computational techniques to support these exams to reduce their evaluation errors. Some authors investigated machine learning traditional methods to classify cervical cells. Kuko and Pourhomayoun [13] proposed a Random Forest approach to classify the cells based on 33 morphological characteristics. Silva et al. [5] evaluated 93 approaches to perform the cell classification. They evaluated the performance with the Support Vector Machine (SVM), k-Nearest Neighbors (k-NN), and Random Forest (RF) algorithms, and 31 sets of characteristics. Isidoro et al. [6] used a SVM to classify images of cervical cells obtained in Pap smear through the extraction of nongeometric characteristics, while Diniz et al. [14] used a hierarchical methodology and geometric characteristics.

In recent years, with the evolution of convolutional methods, several authors have started to study their applicability for image classification. Hussain et al. [7] used the convolutional neural networks AlexNet, VGGNet (VGG-16 and VGG-19), ResNet (ResNet-50 and ResNet-101), and GoogLeNet, as well as their ensemble method, to classify four cervical lesions. Lin et al. [15] proposed a method based on the GoogLeNet, AlexNet, ResNet, and DenseNet convolutional neural networks that combined cell appearance and morphology to perform the classification.

Ghoneim et al. [8] used the Shallow, VGG-16, and CaffeNet architectures to extract characteristics and the Extreme Learning Machine and Autoencoder to classify cervical cells. Li et al. [16] presented an approach based on the Inception-V3 and VGG-16 methods. The methods were constructed, refined, and used in an ensemble version. Mousser and Ouadfel [9] performed a comparative study of the pretrained convolutional neural networks VGG-16, VGG-19, ResNet50, and InceptionV3 to extract cervical cell characteristics. After each convolutional neural network, the authors used a multiLayer perceptron to classify the cells. Sompawong et al. [17] applied Mask R-CNN to detect and classify the cervical cell nuclei.

This work focused on the automatic classification of cervical cells obtained from Pap smear tests using deep learning. The main objective was to maximize the number of true-positive results and minimize the number of false-negative results. Unlike the works above, we sought to use recent convolutional neural networks, the EfficientNets networks, which are state-of-the-art architectures for ImageNet dataset classification. We also considered MobileNet, XceptionNet, and InceptionNetV3 for comparison purposes. We selected these architectures because of their small computational cost, which is advantageous for ensemble methods, such as those proposed in this work.

## 3. Materials and Methods

Several decision support systems have been developed to assist professionals in their tasks and to providing benefits, such as increased efficiency or reducing time and costs [18,19,20,21]. As already mentioned, our methodology is based on the analysis of convolutional neural networks to perform the classification of cell nuclei obtained in images of Pap smears. This task plays a crucial role in creating a decision support tool for cytopathologists.

### 3.1. Dataset

The dataset used in this work is based on the cervical cell classification collection [22] available in the CRIC Searchable Image Database (Available online: https://database.cric.com.br, accessed on 15 February 2021). This dataset contains cervical cell images developed by the Center for Recognition and Inspection of Cells (CRIC).

The Cytology Laboratory of the Pharmacy School generated the dataset images in the Microscopy facility of the Biological Sciences Research Center (NUPEB) of the Federal University of Ouro Preto. The Research Ethics Committee approved this work through the document with Protocol Number 1944523.

The photo documentation was carried out using conventional microscopy in a bright field with a 40× objective and a 10× eyepiece through a Zeiss AxioCam MRc digital camera (Carl Zeiss Ligh Microscope, Göttingen, Germany) coupled to a Zeiss AxioImager Z2 microscope with the Axio Vision Zeiss Software (AxioVision 4.8, Carl Zeiss Ligh Microscope, Göttingen, Germany). and location. Ok, we added. The images are in TIF format, with 1376 × 1020 pixels and a horizontal and vertical resolution of 150 dpi.

Cell classification was performed in consensus with three specialists. First, based on the most recent survey of taxonomic protocols, Pap smear test samples were selected to be used in the CRIC dataset. Three specialists examined the smears under an optical microscope to evaluate the cytomorphological criteria that best represented the classes. After the photo documentation for obtaining the images, the three professionals analyzed, discussed, and selected them to compose the CRIC Cervix collection. The three cytopathologists followed the cervical cell classification protocol. Each cervical cell was classified by selecting the class corresponding to the lesion according to the Bethesda System nomenclature’s cytomorphological criteria and standardized nomenclature, which are currently the most commonly used in this field worldwide.

In addition, they marked the center of the nucleus of each cell. The classification procedure started with an independent classification carried out by the first professional. Then, the second specialist checked the labels. The third professional revised the markings and approved the labels if the three answers were in agreement. Otherwise, the three cytologists reached a consensus to define the final label. The cytopathologists involved in creating the CRIC dataset (from curating the selection of Pap smears to marking cell classifications) had worked with cytological diagnoses for 6, 11, and 20 years. The processes described in their data acquisition showed that the CRIC Cervix dataset was developed with the highest quality, representing the differentiation of cells and classes existing in the Pap smear and reflecting the practical reality in laboratories.

The cervix collection had 400 images obtained from the Pap test smears. The images contained markings representing a position (x,y) located inside the nucleus of the cells that had the classified lesions, which were subsequently classified into six classes: normal (i.e., negative for intraepithelial lesion or malignancy (NILM)); atypical squamous cells of undetermined significance (ASC-US); low-grade squamous intraepithelial lesion (LSIL); atypical squamous cells, cannot exclude high-grade lesion (ASC-H); high-grade squamous intraepithelial lesion (HSIL); squamous cell carcinoma (SCC). The last one was negative for lesions (normal cells), while the others corresponded to a cell with a lesion.

The dataset used in this work is part of the cervical cell classification collection [22] and contains cells from six classes: NILM (862 marks), ASC-US (286 marks), LSIL (598 marks), ASC-H (536 marks), HSIL (874 marks), and carcinoma (SCC-77 marks).

### 3.2. Preprocessing

Several authors, such as [23,24,25], argued that different degrees of cervical lesions correlate with different characteristics of the nucleus. Thus, the nucleus is sufficient for the classification of a cell according to the degree of its lesion.

**Figure 1:**
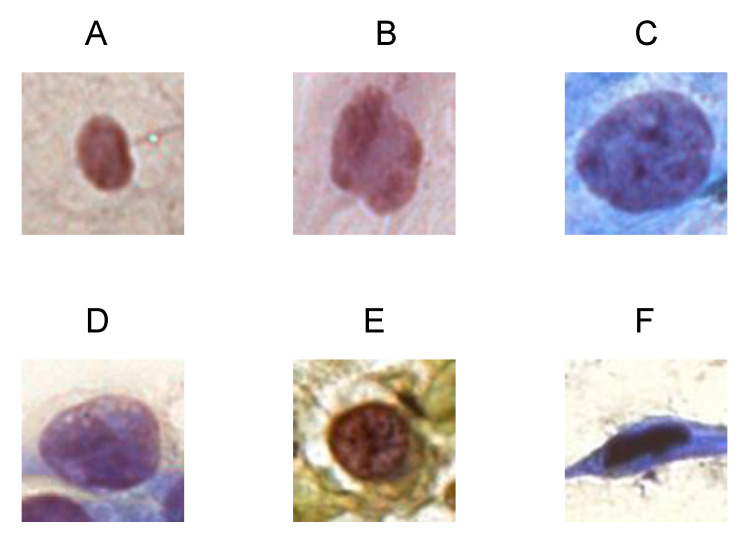
Example of the 90 × 90 cutouts of each class present in the Center for Recognition and Inspection of Cells (CRIC) cervix collection: (**A**) NILM; (**B**) ASC-US; (**C**) LSIL; (**D**) ASC-H; (**E**) HSIL; (**F**) SCC.

This work centered a crop of size m×m on the nucleus demarcated in the dataset. It was decided that m=90 because we empirically identified that this value was large enough to include the entire nucleus in the cropped image and small enough to prevent the appearance of several nuclei in the same cropped image. Figure 1 shows an example of a cutout for each class in the dataset: (A) NILM, (B) ASC-US, (C) LSIL, (D) ASC-H, (E) HSIL, and (F) SCC.

### 3.3. Dataset Division

The classifier generalization is analyzed during the experiments; this refers to how well a model’s learning behaves with new data. We used the holdout method to separate the images into two sets, one for training and the other for testing. During a model’s training, it is common to divide the training set into two other sets: training and validation. The validation set is used to make initial validations during the model’s learning, and the test set is used to measure its generalization [26].

We used 80% of the images to train the convolutional neural networks and 20% for testing. From the training set, 20% of the images were used for validation [27]. Table 1 shows the number of images of each class used in each experiment.

### 3.4. Number of Classes

In addition to the number of classes proposed by the dataset (six classes), this work divided them into two more groups of classes: two-class and three-class groups. The two-class group divided the cell samples into normal and altered. The three-class group split the cell samples into normal, low, and high grades.

The two-class group aimed to rank images based on the presence or absence of lesions. There were only NILM cells in the first class, and in the second class, there were ASC-US, LSIL, ASC-H, HSIL, and SCC lesions, which characterized the altered cell class.

The three-class grouping of lesions was based on the diagnoses and procedures given to patients according to the lesions’ groups. After receiving two negative test results, patients without lesions (normal class) only needed to repeat the test after three years. Patients with low-grade lesions (ASC-US and LSIL) needed to follow up and repeat the exam within six months or one year, depending on the woman’s age. Patients with high-grade lesions (ASC-H, HSIL, and SCC) were indicated for colposcopy and, if necessary, for biopsy.

Finally, the six-class classification was intended to report the type of detected lesion precisely. This classification permits the detailed counting reports of the lesions.

### 3.5. Balance and Data Augmentation

Convolutional neural networks have a large number of parameters to optimize. Thus, they also require a large amount of data for learning. For small datasets, the network may not achieve a desirable generalization power. In these cases, the model is typically overfitted, which is when the model adapts well to the training data but has poor performance for the testing data. To alleviate this problem, data augmentation is used, which is a technique for generating new copies of training data to increase a model’s generality [28].

A relevant aspect of a classification algorithm’s performance is the number of samples and their distribution among the training set classes. When the number of examples is representative, but these examples are unbalanced, classification models that are optimized in terms of the global accuracy tend to create trivial models, which almost always predict the majority class [29].

As already presented, the numbers of images in each class were unbalanced. Therefore, data augmentation was used to balance the data, consequently rearranging their distribution among the training classes and improving the generalization of the data.

To this end, we performed 10 transformations in this study: rotating the original image by 90${}^{{}^{\circ}}$; rotating the original image by 180${}^{{}^{\circ}}$; rotating the original image by 270${}^{{}^{\circ}}$; mirroring the original image; mirroring the image rotated by 90°; mirroring the image rotated by 180${}^{{}^{\circ}}$; mirroring the image rotated by 270${}^{{}^{\circ}}$; adding random noise; adding total-variation noise; and adding noise using a bilateral filter.

We balanced the images of the training/validation datasets according to the number of desired classes. For the six-class balancing, data augmentation was performed based on the largest class, HSIL, which had 699 images. The ASC-H class had 428 images. To balance them, we randomly selected 270 to undergo 1 of the 10 transformations, totaling 698 images. The ASC-US class had 228 images, and each underwent 2 transformations, totaling 684 images. The SCC class had 61 images, and each underwent 10 transformations, totaling 671 images. The LSIL class had 478 images. Of these, 220 were randomly selected and transformed once, totaling 698 images. Finally, the NILM class did not need to be balanced because it already had 689 images.

For the three-class balancing, we found the largest group. The group with normal cells had 689 images, while the low-grade group (ASC-US and LSIL) had 706 images, and the high-grade group (ASC-H, HSIL, and SCC) had 1188. Therefore, because the high-grade group was the largest, the balancing started with that class. We determined that the HSIL (the largest class with 699 images) would be used as a threshold to balance each class in the high-grade group. The balancing of the ASC-H and SCC classes was the same as that for the six classes, resulting in 698 and 671 images, respectively. Thus, the high-grade group had 2068 images.

We also balanced the low-grade and normal cell groups to have approximately 2068 images. Thus, as there were two classes within the low-grade group, the goal of balancing was that each of them had half of the expected images. The first 130 ASC-US images underwent four transformations, and the others (98) underwent three transformations, totaling 1042 images. Furthermore, the first 100 LSIL images underwent two transformations, and the others (378) underwent only one, totaling 1056 images. In the NILM class, each of the 689 images was transformed twice, totaling 2067 images.

Finally, to perform the two-class balancing, we found the largest group. The normal group had 689 images, and the altered group had 1894. Thus, we balanced the altered group of cells first. The largest class (HSIL-699 images) was used as a parameter. The procedure for balancing the ASC-US, LSIL, ASC-H, and SCC classes was the same as balancing the six classes. Thus, we left the group of altered cells with 3452 images. Therefore, for the NILM class to be balanced with the changed class, each image was transformed four times, totaling 3445 images.

Table 2, Table 3 and Table 4 show the numbers of images from each group after the balancing and data augmentation for the two-, three-, and six-class classifications, respectively. Notice that we randomly chose the images and their transformations. The balanced dataset and its division (training, validation, and test) were stored so that all experiments could use the same base to allow comparisons.

### 3.6. Convolutional Neural Network Architectures

Several convolutional neural network architectures have been used to process images [30,31,32], including medical images, as in [8,18,33,34]. For this reason, this study investigated the performance of several convolutional neural network architectures for the classification of cervical cell nuclei obtained in Pap smears. The main architectures investigated were those of the EfficientNets. These architectures focus on improving not only the accuracy but also the models’ efficiency. They are recent proposals and are state of the art for ImageNet dataset classification; in addition, they have small computational costs. Moreover, we selected the MobileNet, XceptionNet, and InceptionNetV3 architectures to compare their performance with EfficientNets. We also chose these architectures because of their low computational costs, which are advantageous for ensemble methods.

The following architectures were considered: EfficientNet (B0 to B6) [35], MobileNet [36], XceptionNet [37], and InceptionNetV3 [38].

#### 3.6.1. MobileNet

**Figure 2:**
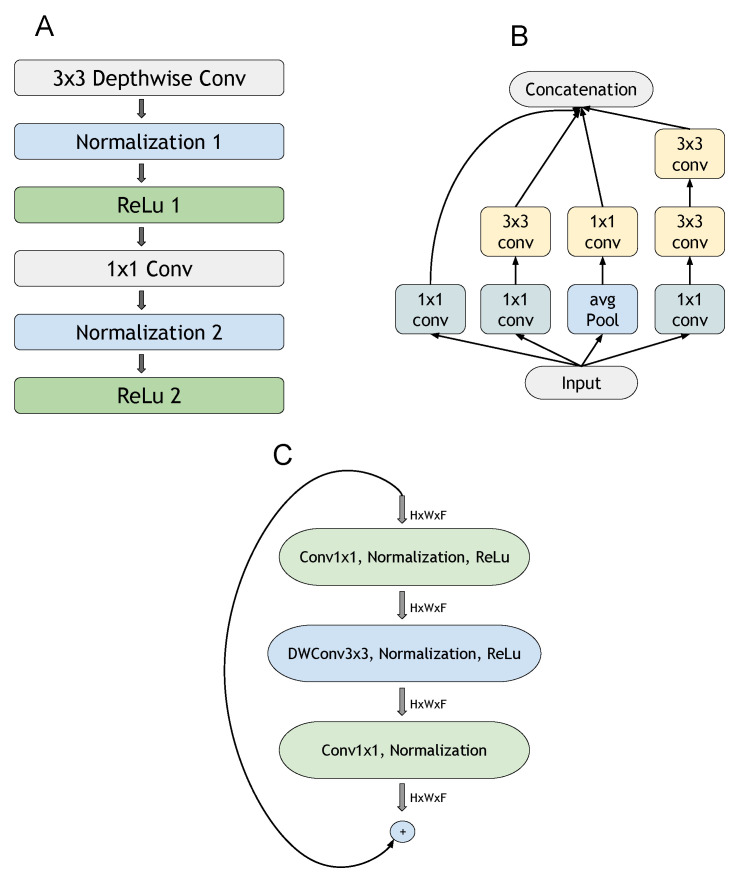
Main blocks of the architectures considered in this work: (**A**) MobileNet block (adapted from [35]); (**B**) InceptionNet block (adapted from [38]); (**C**) EfficientNet block (adapted from [41]).

The MobileNet structure is built with blocks of depthwise and pointwise convolutions as separate layers; see Figure 2. All layers are followed by normalization and ReLU nonlinearity, except the final fully connected layer, which feeds into a softmax layer for classification [36]. The depthwise separable convolution is the key to reducing the model’s footprint.

#### 3.6.2. InceptionNet and XceptionNet

Inception-v3 is a convolutional neural network architecture from the Inception family, which also includes GoogleNet. Blocks were designed for this family to improve the computational costs and other issues [38]. This architecture relies on a 1 × 1 convolution operation and global average pooling to reduce computational costs [39], and it also introduces a multipath block to explore multiple operations on the same input data (see Figure 2). XceptionNets are derived from the Inception architecture; we modified the blocks to use depthwise separable convolution layers with residual connections [37].

#### 3.6.3. EfficientNet

EfficientNets are a family of neural network models designed through a neural architecture search [40] in which the basic building block is the Mobile Inverted Bottleneck Conv Block (MBconv; see Figure 2), which is scaled up in three dimensions: depth, width, and resolution [35]. The neural architecture search algorithm incorporates the reinforcement learning technique to find a baseline network to evaluate the inclusion/exclusion of basic blocks (see Figure 2). Once the baseline network is reached (version B0), the other versions of the network (B1 to B7) are achieved by making the network wider and deeper, including more blocks at the baseline network’s top.

All of the architectures considered were loaded without the last layer and were pretrained with the ImageNet dataset. We removed the last layer to allow the models to adapt to the Pap smear test. The initial layers of the neural networks had essential elements that were present in any image. Therefore, these layers were very similar to those of any problem. We train these initial layers with another dataset and adapt it to the desired problem, making training much faster [28].

After the loaded architecture, we added a flattening layer, a dropout layer of 20%, and a dense layer with the desired number of classes. We applied the flattening layer to transform the matrix from the model into a one-dimensional architecture, which was expected in the posterior layers. The dropout layer was responsible for eliminating neurons during the learning process to avoid overfitting the data. Using empirical tests, we defined the dropout value as 20%. We added the final dense layer to adapt the architecture to solve the classification problem of this work. This layer’s activation function was softmax because it forced the neural network’s output to represent the probability of the data being from one of the defined classes.

The architectures were compiled using the Adam optimizer with a categorical cross-entropy loss function. The metric considered was the recall because it was the most relevant in the problem addressed, as it could measure the number of lesions found. For this problem, it is relevant that all the lesions are found to the women having the appropriate follow-up. Thus, the higher the recall, the better.

**Figure 3:**
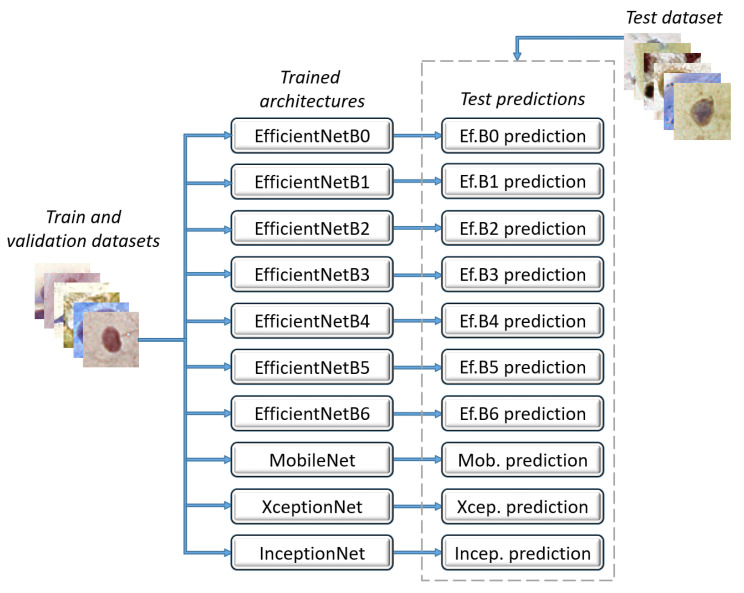
Convolutional neural network architectures.

The architectures were trained and validated with the training and validation datasets, respectively. Then, they received the test dataset to perform their predictions individually, as shown in Figure 3.

### 3.7. Proposed Ensemble

The main idea of the ensemble methodology is to weigh several classifiers and combine them to obtain a classifier that outperforms them individually [42]. Hansen and Salamon [43] added that a necessary ensemble condition is that the classifiers are accurate and diverse.

This methodology was used in this work due to its excellent performance in solving several other classification problems, as introduced by [44,45,46,47].

**Figure 4:**
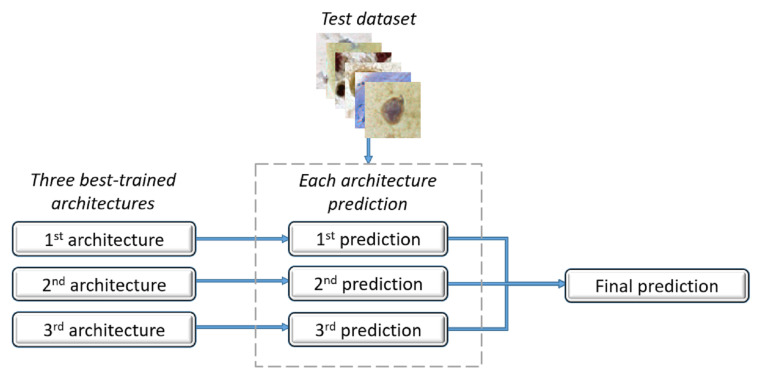
Proposed ensemble.

Once each architecture made its predictions, we composed an ensemble method with the three best architectures regarding the recall values. This method returned the image classifications determined by the majority. In cases of a tie, i.e., each architecture voted for a different class, the vote of the architecture that had the best recall value prevailed. Figure 4 shows the proposed ensemble.

## 4. Experimental Results and Discussion

This section shows and discusses the experiments that were developed to evaluate the proposed methodology. The experiments were performed on a computer with an Intel Core i7-9700F processor with a GPU GeForce RTX 2080, 3 GHz CPU, and 16 GB of RAM, which ran on the Windows 64-bit operating system. The proposed methodology used the Python programming language, version 3.7.9, and the Tensorflow/Keras framework.

In our experiments, we performed shuffle–split cross-validation. We randomly divided the dataset (explained in Section 3.3), balanced it, and performed data augmentation (described in Section 3.5) 10 times, thus generating 10 different training, testing, and validation bases to be used in the ensemble’s construction in order to evaluate the generalizability of our methodology.

### 4.1. Metrics

We used five metrics to measure the proposed methodology’s quality: precision, recall, F1-score, accuracy, and specificity. To calculate them, it was necessary to define the values of true positives (TPs), true negatives (TNs), false positives (FPs), and false negatives (FNs). There were two situations: one in which the test result indicated that the woman had a lesion and another in which she did not. In the first (a result that had a lesion), we defined it as a true positive if the woman had a lesion. However, if she did not have a lesion, we found a false positive. In the second situation (a result that did not have cancer), if the woman had a lesion, the result was a false negative, and if she did not have a lesion, it was a true negative.

Thus, precision (Prec.) measures the test’s capability to detect altered cells only in patients with lesions. Equation (1) presents the formula for precision.

(1) $$Prec.=\frac{TP}{TP+FP}.$$

The recall (Rec.), which was calculated using Equation (2), measures the test’s ability to detect altered cells when they were present.

(2) $$Rec.=\frac{TP}{TP+FN}.$$

The F1-score measure, which is presented in Equation (3), takes the harmonic mean between the precision and recall, indicating the general quality of the proposed methodology.

(3) $$F1-\mathrm{score}=2\times \frac{precision\times recall}{precision+recall}.$$

Accuracy (Acc.) measures the proportion of all tests that gave the results correctly (whether positive or negative) compared to all of the results obtained. The formula used to calculate the accuracy is presented in Equation (4).

(4) $$Acc.=\frac{TP+TN}{TP+FP+TN+FN}.$$

Finally, specificity (Spec.), which is presented in Equation (5), measures the test’s ability to identify not cell lesions when absent; that is, it returns the proportion of people without lesions who had negative test results.

(5) $$Spec.=\frac{TN}{TN+FP}.$$

### 4.2. Results

Table 5 presents the mean results for the precision, recall, F1-score, accuracy, and specificity obtained using cross-validation. These results correspond to the two-class classification of the individual architectures and the proposed ensemble. The best results for each metric are highlighted in bold in this table and in the others. For images classified into two classes, the six individual architectures achieved the same performance: EfficientNetB0, EfficientNetB1, EfficientNetB2, EfficientNetB4, EfficientNetB6, XceptionNet. EfficientNetB1, EfficientNetB2, and EfficientNetB6 were randomly selected as the five best models for performing the ensemble method, and EfficientNetB2 was the tiebreaker decision. Results (Source code available at https://github.com/debnasser/deep-learning-ensemble-jimaging, accessed on 6 July 2021) show that the proposed ensemble outperformed all architectures concerning the five metrics.

**Figure 5:**
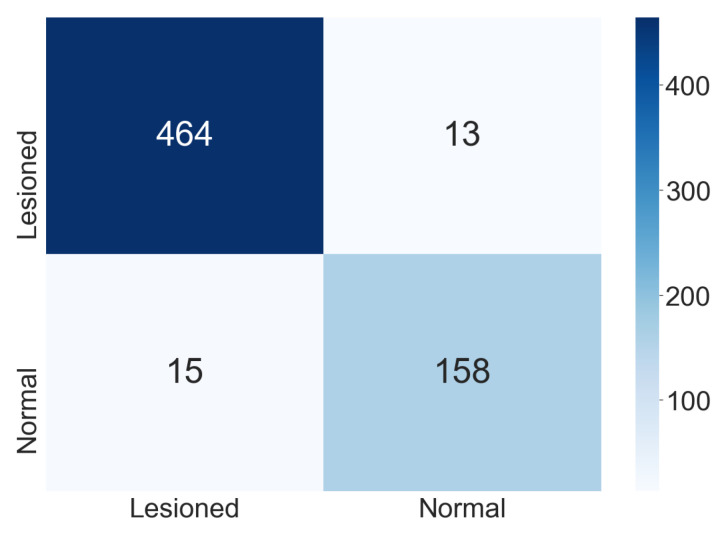
Confusion matrix for the two-class classification.

Figure 5 shows the confusion matrix found in one execution of the ensemble used for the two-class classification. We observed that the classes’ generalization did not change the classification with the confusion matrix and the results presented. Despite aiming the proposed methodology at creating a decision support tool for cytopathologists, the professional will confirm the final classification.

However, when analyzing only the two-class classification, the methodology will not assist the professional by suggesting a diagnosis and an appropriate follow-up for a patient. For this reason, we also analyzed the three-class classification. Table 6 presents the mean results obtained in the cross-validation of the individual architectures and the proposed ensemble for the three-class classification.

For the three-class classification, EfficientNetB2, EfficientNetB4, and EfficientNetB6 were selected to perform the ensemble method, and EfficientNetB6 was the tiebreaker decision. We observe in Table 6 that the proposed ensemble overcame all architectures for the five metrics. EfficientNetB4 and EfficientNetB6 only had the same performance as the ensemble for the specificity metric.

**Figure 6:**
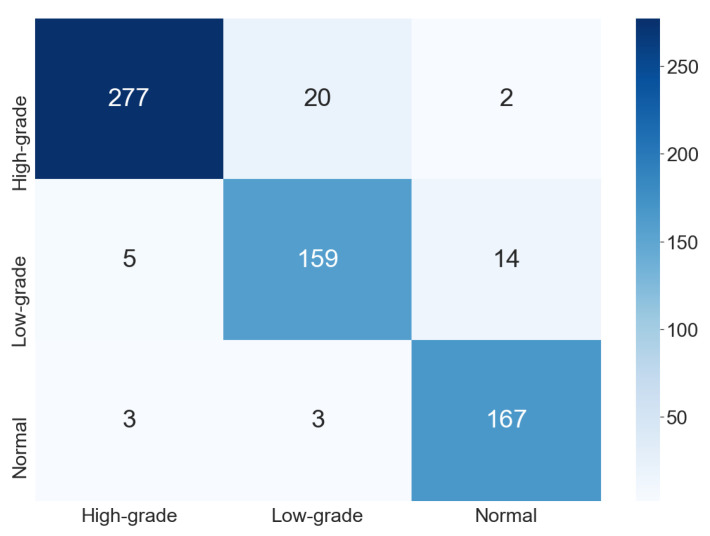
Confusion matrix for the three-class classification.

The confusion matrix shown in Figure 6 refers to one execution of the ensemble for the three-class classification. In comparison with the result for the two-class classification, we can observe that the number of classes is directly related to the classification difficulty. Initially, this behavior was not clear because, despite the common assumption that a smaller number of classes implies an easier problem, the combination of different types of cells in the same class could generate a very distinct class. Thus, a hypothesis would be that this could disturb classification, which was not observed in our results.

An advantage of making the three-class classification is that it suggests the patient’s conduct (in the case of a positive result) and the diagnosis to the cytopathologist. Again, it is relevant to consider that the outcome of the proposed methodology is only a suggestion. The patient’s final diagnosis and follow-up are the responsibility of the cytopathologist; the proposed methodology is only a tool to support their decisions.

We also analyzed the six-class classification. With this classification, we determine the type of lesion presented in an image. Table 7 reports the mean results of the 10 executions of the cross-validation. For this task, we used EfficientNetB1, EfficientNetB2, and EfficientNetB3 to perform the ensemble method. This ensemble method also used EfficientNetB2 as the tiebreaker method because it was one of the architectures that produced the best results in all evaluation metrics. According to our experiments, the proposed ensemble also outperformed all individual architectures. Only EfficientNets B2 and B3 had the same performance as the ensemble for specificity.

**Figure 7:**
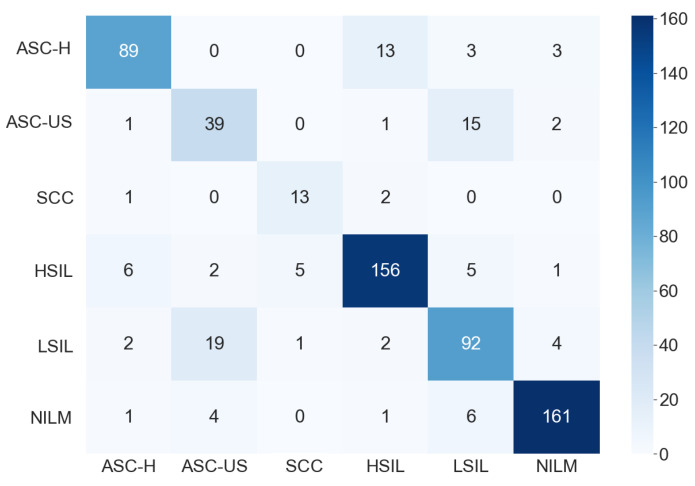
Confusion matrix for the six-class classification.

Figure 7 shows the confusion matrix found by one proposed ensemble for the six-class classification. In this case, we observe that the classes ASC-US and LSIL were quite confused. A relevant feature for differentiate them is the number of occurrences in the smear. When many occurrences are observed, the cells are classified as LSIL; otherwise, they are classified as ASC-US [48]. As this work evaluated cell images individually, it was not possible to use this approach to improve the results, as this would involve evaluating a whole smear. The methodology also disregards relationships between the cropped images. However, both classes (ASC-US and LSIL) lead to the same patient conduct: follow-up and repeating the exam in six months or one year, depending on the patient’s age.

According to these experiments, the EfficientNets stood out among other methods. These neural networks are likely efficient for the problem in question due to their ability to stack multiple layers of the mobile inverted bottleneck (MBConv) blocks combined with squeeze-and-excitation optimization.

To compare the results obtained here with those of other methods found in the literature, the articles proposed by Silva et al. [5] and Isidoro et al. [6] were selected because they used the same dataset as that used in this work. Table 8 compares the results using the precision, recall, F1-score, accuracy, and specificity metrics according to the number of classes covered. According to Equation (1) of the work proposed by Isidoro et al. [6], the authors used precision instead of accuracy. Therefore, we changed the value to the correct one. We inferred the accuracy based on the recall and F1-score. In this table, we also present the classification results for the six-class classification.

In Table 8, we verify that the proposed method outperformed the one presented by Silva et al. [5] in terms of the recall values for the two-class classification. For all of the analyzed metrics, the proposed method was also superior to the method proposed by Isidoro et al. [6] for the two- and three-class classifications.

**Figure 8:**
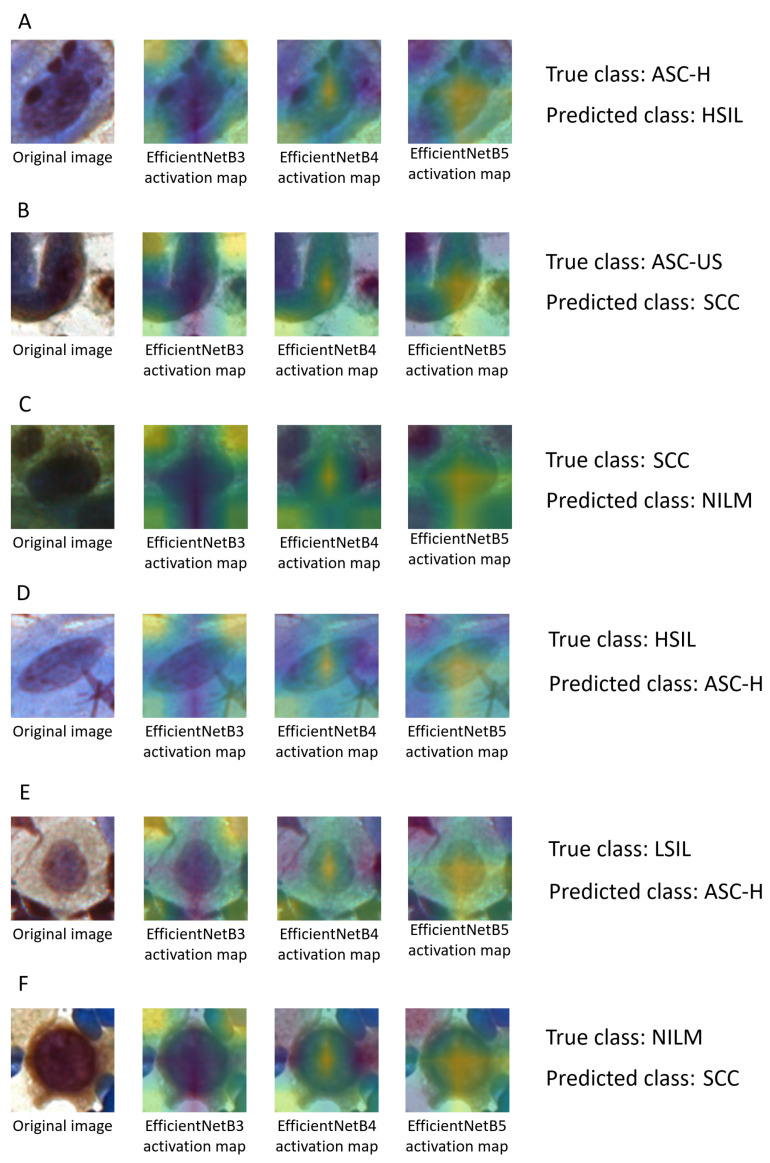
Examples of incorrect classifications: original images, their activation maps according to each architecture used in the ensemble, and their true and predicted classes: (**A**) ASC-H; (**B**) ASC-US; (**C**) SCC; (**D**) HSIL; (**E**) LSIL; (**F**) NILM.

Figure 8 presents the original images and their activation maps according to each architecture used in the ensemble for the six-class classification. All images are examples of incorrect predictions of the classifier. Figure 8 also presents the true and predicted classes.

The cytopathologists of our team performed an analysis of these erroneous classifications. They realized that the information that could contribute to a more assertive classification is related to the morphology of the cytoplasm, such as its area, circularity, and the nuclear-to-cytoplasmic ratio. Therefore, when the selected regions were presented to the cytopathologists, the final diagnoses tended to be precise.

In addition, notice that routine laboratory smear analysis is based on all images and not on isolated images. In real cases, a cytopathologist analyzes several characteristics present in the smear; thus, a set of altered cells is usually necessary to reach a diagnostic decision. Therefore, not identifying a few cells will not jeopardize the result because others will still be identified and will support the cytopathologist’s decision. Thus, as the number of false negatives in this work is relatively low, we expect that the proposed method would be even more helpful for a general analysis of a smear.

## 5. Conclusions

This work proposed an ensemble of algorithms for classifying cells obtained with Pap smear tests based on a study of several architectures of convolutional neural networks. This classification is an essential step in constructing a decision support tool for the analysis of this exam. This tool selects the most likely altered nuclei, which the cytopathologists will manually analyze to diagnose and prognosis. Nowadays, this analysis is performed entirely manually, making it an exhaustive and repetitive task. The proposed method can reduce professionals’ workload and the waiting time for the examination response. The assertiveness of the result can also be increased.

Data augmentation, dropout, and cross-validation strategies were applied to construct a more accurate model, which represents the dataset, avoids overfitting, and improves the performance in the imbalanced dataset.

Considering the precision, recall, F1-score, accuracy, and sensitivity metrics, the proposed ensemble outperformed the methods from the literature in two- and three-class classification. We also introduced the classification results for the six-class classification.

From a biological point of view, the results found here are promising. The best result was obtained for the two-class classification because it was the one that achieved the highest recall value and, consequently, the lowest number of false negatives (maximum of 3%). This is a remarkable result because manual laboratory routines can reach high values (up to 62%) [1,2,3,4]. Furthermore, considering the benefit of three-class classification in suggesting follow-up with a diagnosis, these results can also be beneficial for developing a decision support tool.

As future work, we suggest a further investigation of the whole cell, seeking to improve the results even more.

## Figures and Tables

**Table 1:** The number of images from each class for the training, validation, and testing sets.

Class	NILM	ASC-US	LSIL	ASC-H	HSIL	SCC	Total
Training	551	182	382	342	535	48	2040
Testing	173	58	120	108	175	16	650
Validation	138	46	96	86	164	13	543
Total per class	862	286	598	536	874	77	3233

**Table 2:** The numbers of images from each class after balancing and data augmentation for the training, validation, and testing sets in the two-class classification.

Set	Normal	Altered					Total
	NILM	ASC-US	LSIL	ASC-H	HSIL	SCC	
Training	2756	547	558	558	559	536	5514
Testing	173	58	120	108	175	16	650
Validation	689	137	140	140	140	135	1381
Total	3618	3927					7545

**Table 3:** The numbers of images from each class after balancing and data augmentation for the training, validation, and testing sets in the three-class classification.

Set	Normal	Low-Grade Lesions		High-Grade Lesions			Total
	NILM	ASC-US	LSIL	ASC-H	HSIL	SCC	
Training	1653	833	844	558	559	536	4983
Testing	173	58	120	108	175	16	650
Validation	414	209	212	140	140	135	1250
Total	2240	2276		2367			6883

**Table 4:** The numbers of images from each class after balancing and data augmentation for the training, validation, and testing sets in the six-class classification.

Set	NILM	ASC-US	LSIL	ASC-H	HSIL	SCC	Total
Training	551	547	558	558	559	536	3309
Testing	173	58	120	108	175	16	650
Validation	138	137	140	140	140	135	830
Total	862	742	818	806	874	687	4789

**Table 5:** Results for the two-class classification. The best results for each metric are highlighted in bold.

Architecture	Prec.	Rec.	F1-Score	Acc.	Spec.
EfficientNetB0	0.95	0.95	0.95	0.95	0.95
EfficientNetB1	0.95	0.95	0.95	0.95	0.95
EfficientNetB2	0.95	0.95	0.95	0.95	0.95
EfficientNetB3	0.94	0.94	0.94	0.94	0.94
EfficientNetB4	0.95	0.95	0.95	0.95	0.95
EfficientNetB5	0.94	0.94	0.94	0.94	0.94
EfficientNetB6	0.95	0.95	0.95	0.95	0.95
MobileNet	0.94	0.94	0.94	0.94	0.94
XceptionNet	0.95	0.95	0.95	0.95	0.95
InceptionNetV3	0.92	0.92	0.92	0.92	0.92
Ensemble	**0.96**	**0.96**	**0.96**	**0.96**	**0.96**

**Table 6:** Results for the three-class classification. The best results for each metric are highlighted in bold.

Architecture	Prec.	Rec.	F1-Score	Acc.	Spec.
EfficientNetB0	0.92	0.92	0.92	0.94	0.96
EfficientNetB1	0.92	0.92	0.92	0.95	0.96
EfficientNetB2	0.92	0.92	0.92	0.95	0.96
EfficientNetB3	0.91	0.91	0.91	0.94	0.96
EfficientNetB4	0.93	0.93	0.93	0.95	**0.97**
EfficientNetB5	0.92	0.92	0.92	0.95	0.96
EfficientNetB6	0.93	0.93	0.93	0.95	**0.97**
MobileNet	0.91	0.91	0.91	0.94	0.95
XceptionNet	0.92	0.92	0.92	0.95	0.96
InceptionNetV3	0.83	0.83	0.83	0.89	0.92
Ensemble	**0.94**	**0.94**	**0.94**	**0.96**	**0.97**

**Table 7:** Results for the six-class classification. The best results for each metric are highlighted in bold.

Architecture	Prec.	Rec.	F1-Score	Acc.	Spec.
EfficientNetB0	0.82	0.82	0.82	0.94	0.96
EfficientNetB1	0.82	0.82	0.82	0.94	0.96
EfficientNetB2	0.83	0.83	0.83	0.94	**0.97**
EfficientNetB3	0.83	0.83	0.83	0.94	**0.97**
EfficientNetB4	0.81	0.81	0.81	0.94	0.96
EfficientNetB5	0.82	0.82	0.82	0.94	0.96
EfficientNetB6	0.82	0.82	0.82	0.94	0.96
MobileNet	0.77	0.77	0.77	0.92	0.95
XceptionNet	0.80	0.80	0.80	0.93	0.96
InceptionNetV3	0.55	0.55	0.55	0.85	0.91
Ensemble	**0.85**	**0.85**	**0.85**	**0.95**	**0.97**

**Table 8:** Comparison with the methods from the literature. The best results for each metric are highlighted in bold.

Method	Classes	Prec.	Rec.	F1-Score	Acc.	Spec.
Proposed method	two	**0.96**	**0.96**	**0.96**	**0.96**	**0.96**
Proposed method	three	**0.94**	**0.94**	**0.94**	**0.96**	**0.97**
Proposed method	six	**0.85**	**0.85**	**0.85**	**0.95**	**0.97**
k-NN [5]	two	-	0.95	-	-	-
RF [5]	two	-	0.94	-	-	-
SVM [5]	two	-	0.90	-	-	-
SVM [6]	two	0.90	0.92	0.91	0.90	0.88
SVM [6]	three	0.86	0.95	0.90	0.85	0.78

## Data Availability

The dataset used in this work is available at CRIC Searchable Image Database (Available online: https://database.cric.com.br, accessed on 15 February 2021).

## Abbreviations

The following abbreviations are used in this manuscript:

Acc.	Accuracy
ASC-US	Atypical Squamous Cells of Undetermined Significance
ASC-H	Atypical Squamous Cells, cannot exclude HSIL
BHS	Brazilian Health System
CRIC	Center for Recognition and Inspection of Cells
FN	False Negative
FP	False Positive
HSIL	High-Grade Squamous Intraepithelial Lesion
k-NN	k-Nearest Neighbors
LSIL	Low-Grade Squamous Intraepithelial Lesion
NILM	Negative for Intraepithelial Lesion or Malignancy
Prec.	Precision
Rec.	Recall
RF	Random Forest
SCC	Squamous Carcinoma
Spec.	Specificity
SVM	Support Vector Machine
TN	True Negatives
TP	True Positives
